# Agreement of in-ear temperature to core body temperature measures during invasive whole-body cooling for hypothermic circulatory arrest in aortic arch surgery

**DOI:** 10.1038/s41598-024-77237-5

**Published:** 2024-11-11

**Authors:** Jonas Langenhorst, Aaron Benkert, Sven Peterss, Matthias Feuerecker, Tatjana Scheiermann, Patrick Scheiermann, Matthias Witte, Aaron Benkert, Andreas Bayer, Stephan Prueckner, Maximilian Pichlmaier, Roman Schniepp

**Affiliations:** 1 Present Address: Institut für Notfallmedizin und Medizinmanagement, (INM), LMU Klinikum, LMU München, Germany; 2 Department of Neurology, LMU Klinikum, LMU München, Germany; 3 Department of Anesthesiology, LMU Klinikum, LMU München, Germany; 4 Department for Cardiac Surgery, LMU University Hospital, LMU Klinikum, LMU München, Germany

**Keywords:** Circulatory arrest, Therapeutic hypothermia, Core body temperature, In-ear temperature, Aortic arch, Diagnostic markers, Vascular diseases, Aortic diseases

## Abstract

Targeted temperature management (TTM) with therapeutic hypothermia (TH) during aortic arch surgery requires valid estimations of core body temperature. The ear canal and epitympanic region might be an easy-to-assess, noninvasive site for the read-out of supra-aortic, cerebral temperature. This observational cohort study comparatively investigated in-ear temperature and different core body temperature (cBT) measurements during TTM/TH for moderate hypothermic circulatory arrest (mHCA) in aortic arch surgery. In total 24 patients (mean age of 56.8 ± 17.5 years; six females) were measured using infrared-thermography of the epitympanic region (BT_tym_), thermistor-based measurements at the esophagus (BT_eso_; gold standard), at the ear canal (BT_ear_), at the nasopharynx (BT_nas_), in the bladder (BT_ves_), and in the rectum (BT_rec_). The data analysis comprised absolute agreement (AA), bias, intraclass correlation coefficient (ICC), and limit of agreement (LoA). The results revealed high AAs of BT_tym_, BT_ear_, BT_nas_ in reference to BT_eso_ (biases 0.3–0.6 °C), with also excellent ICCs > 0.9. BT_ves_ and BT_rec_ showed lower AAs, higher biases of + 2.5 °C to 3.1 °C with moderate ICCs during mHCA. In the phases of rapid temperature changes, the biases and LoAs were higher throughout all BT measurements. Herein, BT_tym_ performed best of all measurement sites. The study informs about the BT dynamics at different body sites during the mHCA procedure. It supports the approach of using minimally invasive in-ear techniques to estimate core body temperature in an intrahospital TTM/TH setting of mHCA.

## Introduction

Targeted temperature management (TTM) with therapeutic hypothermia (TH) is a fundamental part of the neuroprotective strategy during aortic arch surgery^1^. Procedures extending into the arch per definition require circulatory arrest (CA). The vulnerability of the brain to hypoxemia determines the duration and potential harmfulness of the iatronegic CA procedures. The induction of hypothermia to a targeted core body temperature (cBT) of 22–26 °C prior to CA^2^ enhances the ischemic tolerance of the central nervous system by multiple mechanisms^3^. Thus, TTM/TH with rapid change rates and moderate hypothermic circulatory arrest (mHCA) is a clinical standard procedure for aortic arch surgery. The 2021 ERC guidelines also recommend TTM/TH with a higher cBT target range of 32–34 °C for neuroprotection after out-of-hospital cardiac arrest^4^.

TTM/TH procedures demand for continuous and accurate cBT monitoring with a desired read-out of cerebral temperature. With the rapid temperature changes during cooling and rewarming phases for mHCA, the cBT measurement procedures are supposed to show immediate and fast dynamic behavior. The most accurate methods for this purpose is the measurement of central blood temperature at the pulmonary artery, but limited by its high degree of invasity. The measurement of tissue temperature in the middle part of the esophagus is therefore regularly chosen as clinical standard method for TTM/TH monitoring in aortic arch surgery^2^. Monitoring of rectal or bladder cBT is less invasive. However, cBT changes recorded in the bladder or in the rectum are considered slower in response and with inferior temporal dynamics of BT^4^.

The external ear canal as a possible measurement site enables further potentials for body temperature (BT) monitoring. The easy accessibility of the ear canal and the development of wearable in-ear sensors, nowadays, enable a minimally invasive and continuous monitoring of epitympanic or aurical BT. The proximity of the (epi-) tympanum to intracranial arteries and the hypothalamus potentially allows the estimation of cBT^5^, respectively cerebral temperature^6^. Additionally, the minor sympathic influence of its arteries may lead to a preserved vascularization of the epitympanum and ear canal during centralization induced by hypotonia or hypothermia^7^.

Recent intra-hospital investigations demonstrated a satisfactory concordance between epitympanic BT and cBT (derived from the pulmonary artery) in mild TTM/TH^8^ and mHCA^9^, with no evaluation of the clinical standard of esophageal BT in either study. Nonetheless, investigations with different technical or procedural specifications have reported contradictory findings^10^.

To generate comparative data, this study systematically investigates cBT measurements at various sites during the whole mHCA procedure while aortic arch surgery. The primary objective is to examine the concordance and limits of agreement of different body temperature measurements in reference to the clinical gold standard of esophageal cBT. The second objective is the investigation of the temporal dynamics of different cBT measures during rapid temperature gradients in the TTM/TH procedure.

## Methods

### Participants

Sample size calculation (MedCalc® 23.0.2) was performed with the assumptions α = 0.05, β = 0.20 and an expected mean difference of 1.0 ± 0.8 °C between the different measures. The minimum number of participants for repeated measures Bland-Altmann-procedures was n = 17, for repeated measures ANOVA it was n = 24.

In total 24 patients (mean age of 56.8 ± 17.5 years; 6 females) were recruited as part of the in-ear temperature and perfusion monitoring during therapeutic hypothermia study (in-ear-TEMPERATURE; DRKS-ID: DRKS00032338) at the LMU University Hospital, Ludwig Maximillian University Munich, Bavaria, Germany. Inclusion criteria were (I) an age above 18 years, and (II) a planned replacement of the proximal arch of the aorta with mHCA. Exclusion criteria were (I) the presence of clinical signs of inflammation of the ear canal, (II) an auditory canal obstruction (anatomic/ foreign material), and (III) a history of tympanic plastic surgery. Each participant provided written informed consent before participation. Ethical approval for this study (Ethical Committee N° 22–0322) was provided by the Ethical Committee of the LMU University Hospital, Munich, Germany in agreement with the Declaration of Helsinki.

### Study procedures and recordings

Following induction of anesthesia, thermistor probes (Ruesch Rectal/Temperature Sensor, Teleflex Medical, Ireland) were inserted into the bladder, rectum, middle esophagus (standardized placement 0.35 m away from teeth row), and nasopharynx (standardized placement 0.20 m away from nasal apertura). Continuous temperature data was assessed with 0.1 Hz. A commercially available, medical device ear canal sensor (Cosinuss° cmed° alpha^®^) was inserted into the right ear without further insulation of the ear. The Cosinuss° cmed° alpha^®^ sensor includes both a thermistor and an IR-thermography for direct mode, in-ear measurement of temperature (0.1 Hz). The IR-thermography directs towards the tympanum, thus delivering BT data of epitympanic tissue. The thermistor of the Cosinuss° cmed° alpha^®^ records BT of the external ear canal tissue. The BT measurements from the thermistor probes and the in-ear sensor were calibrated via the blackbody and water bath procedure according to the ASTM International standard^11^. After the surgery, the thermistor probes and the ear sensor were removed, leaving the thermistor in the bladder for clinical routine at the ICU.

### Hypothermia procedure during surgery

During anesthesia induction and transport to the operation room, no standardized TTM/TH procedure was performed. A possible passive reduction of BT during this period was tolerated, when BT maintained > 35 °C (Table 1, phase 1). In the following sequence, an standard procedure for mHCA at the cardiac surgery unit included a target cBT measured in the bladder < 27 °C (phase 3) and a specific cooling and rewarming protocol (phases 2 and 4). It was defined by a maximum temperature difference of 6 °C (for phase 2) and 3°C (for phase 4) between perfusate delivered by the heart–lung machine (HLM) and the drained venous blood temperature. The bladder was deliberately chosen as the measurement site for target cBT to ensure thorough cooling of all tissue regions of the body at the initiation of circulatory arrest. Further institutional standards of patient management during aortic arch surgery are described elsewhere^12^ and can be reviewed in the supplemental data. Table 1 highlights the definitions of the starting and ending time points of the mHCA procedure. Phase 1 involved the preparation at normothermic conditions; phase 2 comprised the cooling process; phase 3 included hypothermic circulatory arrest, while phase 4 entailed the rewarming process.

**Table 1:** Procedural definitions of the four TTM/TH phases.

Phase	Name	Start	End
1	Preparation	Start of preparation	Start of cooling process/HLM
2	Cooling process	Start of cooling process/ HLM	BT_ves_ < 27 °C
3	Hypothermic circulatory arrest (HCA)	BT_ves_ < 27 °C	start of warming process/HLM
4	Rewarming process	Start of warming process/HLM	BT_ves_ ≥ 36 °C

Temporal divisions of the study with procedural definitions of starting and ending points.

BT: body temperature; HLM: heart–lung machine; ves: vesicular.

### Data processing and analysis

BT data was recorded with a sampling rate of 0.1 Hz and resampled for 1 min intervals. Herein, the mean and standard deviation (SD) of BT were calculated for the bladder (BT_ves_), for the rectum (BT_rec_), for the nasopharynx (BT_nas_), for the esophagus (BT_eso_), for the epitympanic region (BT_tym_), and for the external ear canal (BT_ear_).

The time stamps for the Draeger^®^ and Cosinuss°^®^ systems were synchronized manually by setting the system clocks of both devices with a double check via manual light tapping of the ear sensor accelerometer (related to the system clock of the Draeger^®^ monitoring device).

The Kolmogorov–Smirnov test assessed the distribution of the derived parameters followed by a descriptive analysis for the absolute temperature values. A repeated measure ANOVA (rmANOVA) with Bonferroni’s post-hoc analysis was utilized to calculate the f-value (F) and p-value (p) for the independent factor “measurement site”. Additionally, Bland–Altman procedures (for repeated measures) with limits of agreement (LoA) and intraclass correlation coefficients (ICC, two-way mixed model, absolute agreement) were calculated with BT_eso_ as the reference for all BT measures. The ICC was evaluated according to the following standards: low (< 0.39); moderate (0.40–0.59); high (0.60–0.79); and excellent (≥ 0.80)^13^. Results were considered significant at p < 0.05. Statistical analysis was performed using SPSS (Version 29.0; IBM Corp., Aronk, NY).

## Results

### Characteristics of the study cohort and surgery procedures

Demographic characteristics and health variables were computed and are presented in Table 2. The mean age of the cohort was 56.8 ± 14.8 years, six of the 24 patients were female. All patients underwent mHCA according to the standard operation procedure for the replacement of the proximal arch of the aorta.

**Table 2:** Patient characteristics and surgery procedures.

ID	Sex	Age	Diagnoses	AR	MR	AAR	Others	Duration of surgery
1	m	77	TAA			X		2:46
2	m	45	AI, TAA	X		X		4:17
3	f	55	AI, TAA	X	X	X		4:42
4	m	56	AI, TAA	X		X		3:01
5	m	75	AI, TAA			X		2:52
6	f	80	AI, TAA	X		X		3:46
7	m	50	AI, TAA	X	X	X		3:31
8	m	52	AI, TAA	X	X	X		3:16
9	m	59	AI, TAA	X		X		2:51
10	m	53	AI, TAA	X	X	X		3:51
11	m	75	AI, TAA	X		X		3:21
12	m	49	AI, TAA	X	X	X		5:12
13	f	70	AI, TAA			X		3:04
14	m	61	AI, TAA	X		X		3:59
15	m	62	AS, CHD, TAA,	X		X		4:31
16	m	76	AI, TAA, PFO	X		X	PFO occlusion	4:59
17	m	31	TAA, PFO	X		X	PFO occlusion	3:49
18	m	39	AI, TAA	X	x	X		3:37
19	f	71	TAA			X		3:00
20	f	28	AI, TAA	X		X		3:56
21	m	59	AI, TAA	X		X		3:37
22	m	29	AI, TAA	X		X		4:17
23	f	54	AS, TAA	X		X		4:07
24	m	57	AS, TAA	X		X		3:33

Patient characteristics and surgery procedures of the included participants.

AAR: aortic arch replacement; AI: aortic valve insufficiency; AR: aortic valve reconstruction/replacement; AS: aortic valve stenosis, f: female; hh: hours, m: male; mm: minutes; MR: mitral valve reconstruction/replacement; PFO: persistent oval foramen, TAA: thoracic aortic aneurysm.

The mean duration of the surgical procedure for participants was 225 ± 41 min, with phase 1 (P1) of 55 ± 22 min, phase 2 (P2) of 46 ± 12 min, phase 3 (P3) of 25 ± 7 min, and phase 4 (P4) of 91 ± 21 min.

### ANOVA model of absolute body temperature measurements

For all time sections of the study and for all measurement sites, normal distribution was evident for BT (all p > 0.05 in the Kolmogorov–Smirnov test).

The mean BT_eso_ was 35.5 ± 0.5 °C during P1 and 23.4 ± 1.1 °C during P3. The mean change of BT_eso_ was 0.4 ± 0.1 °C/min for P2, respectively 0.4 ± 0.1 °C/min for P4 (Table 3). The rmANOVA model revealed a significant effect of the variable “measurement site” on the detected BT during P3 (F6, 23) = 2.4, p < 0.05), on the change of BT (ΔBT) during P2 (F6, 23 = 6.4, p < 0.05), and on ΔBT during P4 (F6, 23 = 10.2, p < 0.01) (Table 3).

**Table 3:** Summary of the rmANOVA model for BT measurements.

Phase	Duration [min]	BT_eso_ [°C]	BT_tym_ [°C]	BT_ear_ [°C]	BT_nas_ [°C]	BT_ves_ [°C]	BT_rec_ [°C]	p-value
Static phases		Absolute body temperature/mean ± SD						
1	55 ± 22	35.5 ± 0.5	35.5 ± 0.5	35.6 ± 0.8	35.5 ± 0.5	36.0 ± 0.5	36.2 ± 0.5	n.s.
3	25 ± 7	23.4 ± 1.1	23.1 ± 1.1	23.2 ± 1.0	23.4 ± 1.1	*26.0* ± *1.1*	*26.4* ± *1.4*	0.031
Dynamic phases		Δ of body temperature °/min/mean ± SD						
2	46 ± 12	0.4 ± 0.1	0.4 ± 0.1	0.4 ± 0.1	0.4 ± 0.1	*0.3* ± *0.1*	*0.2* ± *0.1*	0.013
4	91 ± 21	0.3 ± 0.1	0.3 ± 0.1	0.3 ± 0.1	0.3 ± 0.1	*0.1* ± *0.1*	*0.1* ± *0.1*	0.004

The ANOVA model reveals significant differences in absolute BT during the hypothermic circulatory arrest and in ΔBT during cooling and rewarming phases.

Values in italic indicate significant differences in the post-hoc pairwise comparisons (compared to BT_eso_).

BT: body temperature; C: Celsius; min: minute.

For absolute BT at P3, Bonferroni posthoc tests showed significantly higher values of BT_ves_ and BT_rec_ compared to the BT_eso_ (p < 0.01 for both pairs). The duration of T3 did not significantly influence these positive offsets (z = − 0.82, p = 0.089). There were no significant differences between BT_eso_, BT_tym_, BT_ear_, and BT_nas_ in pairwise comparisons.

During both dynamic phases (P2 & P4), Bonferroni posthoc tests showed significantly lower ΔBTs for BT_ves_ and BT_rec_ in comparison to BT_eso_ (p < 0.01 for both pairs).

### Intraclass correlation coefficient (ICC), temperature differences, and Bland-Altmann plots

Significant ICC values (in reference to BT_eso_) were evident for all phases of the study. The ICC values ranged from -0.067 to 0.986 (p’s < 0.001) (Table 4), with lowest values for BT_ves_ and BT_rec_. In contrast, the ICC values for BT_tym_, BT_ear_, and BT_nas_ were consistently > 0.8 across all four phases of the study, indicating excellent correlations between these BTs (Table 4).

**Table 4:** Summary of the ICC model for BT measurements.

**Phase**	BT_tym_		BT_ear_		BT_nas_		BT_ves_		BT_rec_	
	ICC	F|p	ICC	F|p	ICC	F|p	ICC	F|p	ICC	F|p
Static phases										
1	**0.825**	5.9|0.000	**0.814**	5.7|0.000	**0.865**	7.4|0.000	**0.591**	5.7|0.000	**0.462**	5.2|0.000
3	**0.923**	16.8|0.000	**0.861**	7.0|0.000	**0.918**	13.1|0.000	**-0.067**	0.8|n.s	**0.160**	2.0|0.000
Dynamic phases										
2	**0.983**	58.9|0.000	**0.975**	47.6|0.000	**0.959**	26.0|0.000	**0.753**	9.8|0.000	**0.719**	9.3|0.000
4	**0.986**	72.9|0.000	**0.979**	57.6|0.000	**0.975**	40.7|0.000	**0.879**	12.4|0.000	**0.861**	11.2|0.000

Intraclass correlation coefficients were calculated in references to BT_eso_. Minimal absolute BT differences and highest values for ICC were found for BT_tym_ and BT_nas_.

BT: body temperature; C: Celsius, ICC: intraclass correlation coefficient.

Significant values are in bold.

The mean BT differences (biases) of BT_tym_ and BT_nas_ compared to BT_eso_ were less than 0.5 °C. The lowest biases were present for BT_tym_ (here fore: biases < 0.3 °C, LoAs < 2.3 °C). For BT_ear,_ temperature biases were slightly higher in the static phases of P1 and P3. During the dynamic phases P2 and P4, the biases were smaller compared to BT_nas_ (Table 5), although the rmANOVA model did not reveal significant differences for these three BT measures.

**Table 5:** Body temperature biases and limits of agreement.

Phase	BT_tym_		BT_ear_		BT_nas_		BT_ves_		BT_rec_	
	Diff [°C]	LoA [°C]	Diff [°C]	LoA [°C]	Diff [°C]	LoA [°C]	Diff [°C]	LoA [°C]	Diff [°C]	LoA [°C]
Static phases										
1	− 0.02 ± 0.43	**0.77**	− 0.05 ± 0.32	**1.6**	0.04 ± 0.38	**0.77**	0.55 ± 0.37	**0.75**	0.73 ± 0.38	**0.76**
3	− 0.28 ± 0.51	**1.00**	− 0.26 ± 0.80	**1.57**	− 0.16 ± 0.55	**1.09**	*2.56* ± *1.64*	***3.21***	*3.02* ± *1.49*	***3.02***
Dynamic phases										
2	0.11 ± 1.17	**2.28**	0.54 ± 1.30	**2.55**	0.45 ± 1.71	**3.45**	*3.53* ± *2.55*	***4.21***	*3.80* ± *2.52*	***4.45***
4	0.07 ± 1.04	**2.09**	− 0.06 ± 1.34	**2.64**	− 0.21 ± 1.42	**3.80**	− *1.74* ± *2.69*	***4.31***	− *1.85* ± *2.29*	***4.55***

Results for the absolute temperature difference and LoAs of the different BT measurement sites in reference to BT_eso_. Italic values indicate significant differences in the post-hoc pairwise comparisons.

C: Celsius; LoA: Limits of Agreement.

Significant values are in bold.

**Fig. 1:**
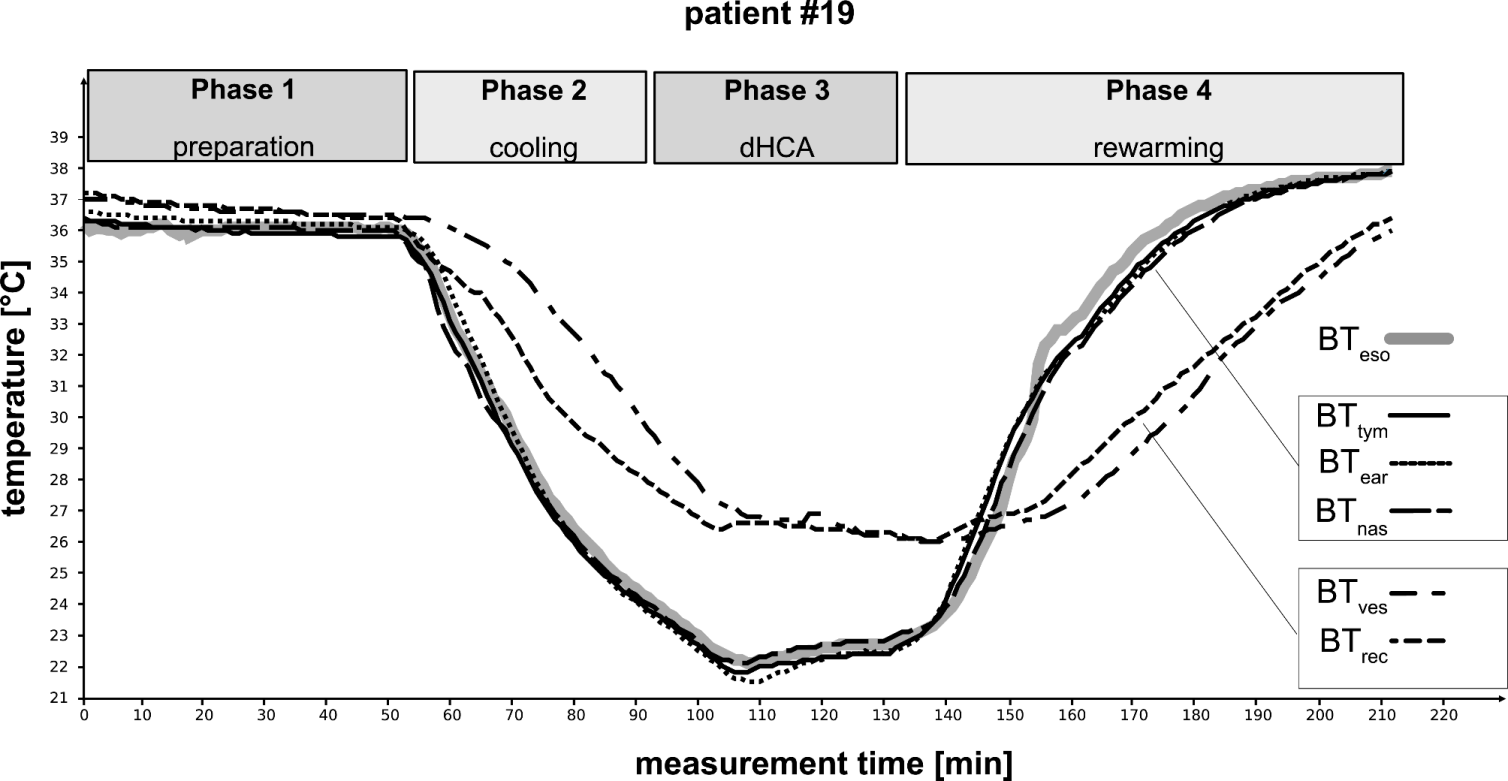
Single patient comparative BT measurement results. Comparative BT data of patient #19 for all study phases. BT: body temperature; C: celsius; CA: cardiac arrest.

RmANOVA with posthoc comparisons revealed significantly higher biases of BT_ves_ and BT_rec_ compared to BT_tym_, BT_ear_, and BT_nas_ (all p < 0.01; Bonferroni posthoc analysis) (Table 5). These findings were evident for P2, P3, and P4 of the study (single patient plot: Fig. 1).

**Fig. 2:**
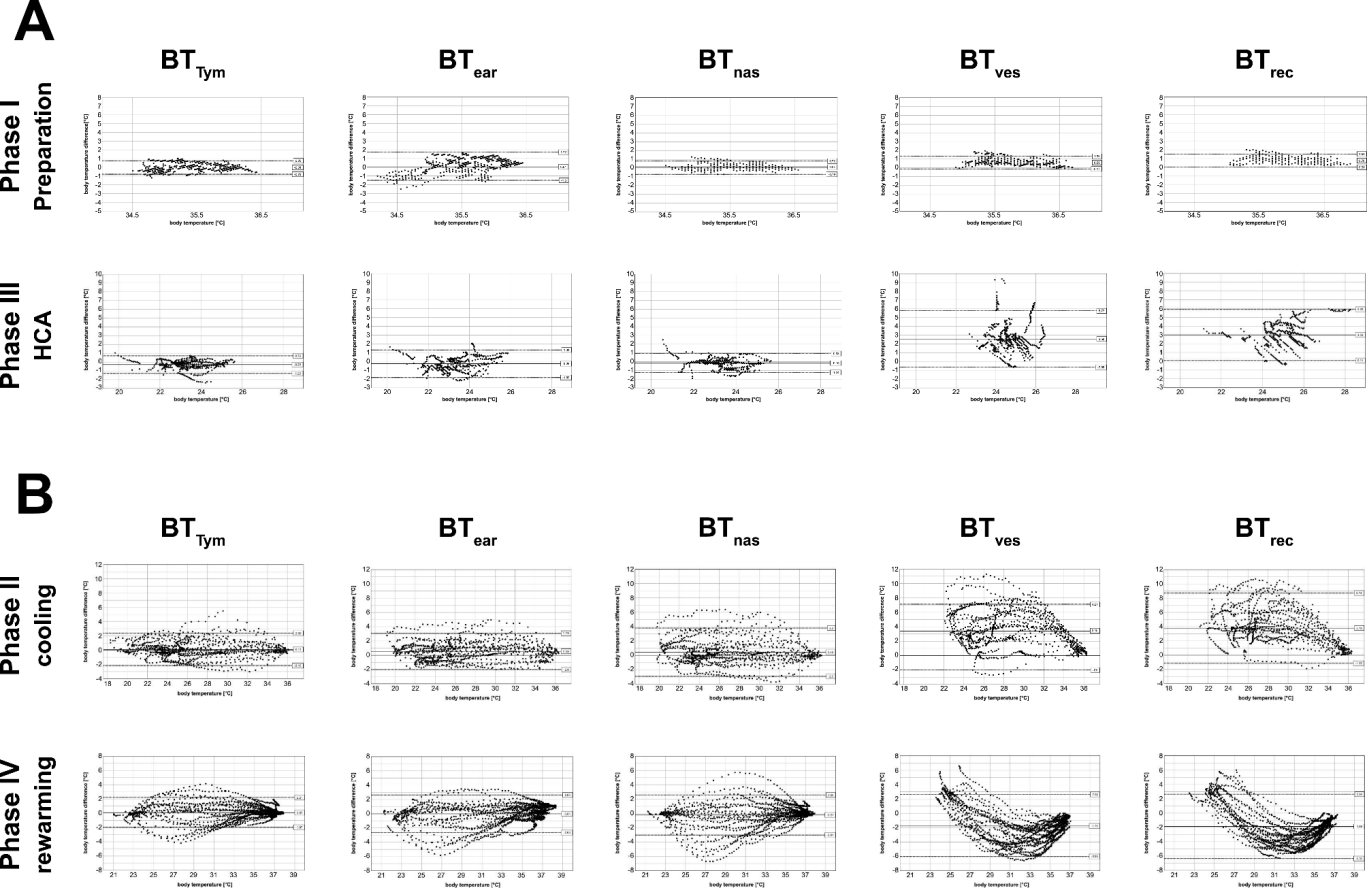
Bland-Altmann plots of all BT measurement sites and all study phases. Bland–Altmanns plots for the different BT measurement site references to BT_eso_. Section A: static BT phases (normothermia and hypothermia), Section B: dynamic BT phases (cooling and rewarming). x-axis: (BT + BT_eso_)/2 in [°C]; y-axis: BT- BT_eso_. BT: body temperature; C: Celsius; HCA: hypothermic cardiac arrest; LoA: Limits of Agreement.

Correspondingly, the LoAs of BTs at all measurement sites showed narrow ranges during normothermia, with the lowest values for BT_tym_ and BT_nas_ (both 0.77 °C). The LoAs for BT_ves_ and BT_rec_ were significantly larger than those of supra-aortic regions, especially during P2, P3, and P4 (all p < 0.01; according to Bonferroni post-hoc analysis). The rapid changes of BT during P2 and P4 induced higher LoAs also for BT_tym_, BT_ear_, and BT_nas_, compared to the phases P1 and P3 (for details: Table 5, Fig. 2). BT_tym_ revealed preferable LoA values for all study phases. The BAP comprised the overall range of all measured cBT values of the cohort, where the extremities of the range mainly represent the static conditions P1 and P3.

## Discussion

Core body temperature measurements at the middle part of the esophagus or in the pulmonary artery are the gold standards for accurate, intraoperative monitoring. Specific characteristics of the TTM/TH procedures for mHCA induce a more elaborate cBT monitoring strategy. Fast temperature gradients during HLM circulation require an immediate responsiveness of the cBT estimation^2^. Moreover, there is a specific interest in the monitoring of cerebral body temperature. Thus, scientific efforts have been made to evaluate non-invasive temperature estimations in supra-aortal regions^14^, such as the measurement of the tympanic BT^15^. This study delivers BT data at various body sites allowing comparisons between supra-aortal circulation regions and lower-body measurement sites.

The study’s main findings are as follows:

I. There is a high absolute agreement for body temperature measurements of the esophagus, epitympanic region, external auditory canal, and nasopharynx during normothermia and mHCA. The body temperature measurements in the bladder and rectum exhibit significantly higher biases during mHCA.
II. The fast temporal dynamics during the cooling and rewarming phases induce wider limits of agreement for all body temperature estimations. Best absolute agreements, intraclass coefficient correlations and limits of agreements are present for the epitympanic BT measurements.

### Body temperature estimation in regions of supra-aortic circulation

All three supra-aortic measurement sites delivered BT data with good absolute agreements and biases ranging from < 0.3 °C (epitympanic) to < 0.6 °C (nasopharynx, ear canal). These findings suggest similar temperature homeostasis in tissues of supra-aortic circulation and the mediastinal tissues, which is in accordance with recent studies using pulmonary artery blood temperature as the gold standard^8,16^. Further, supra-aortic BT measurement appears to be also valid during the process of mHCA, which complements studies in procedures of mild TTM/TH after cardiac arrest^17^.

Only minor, non-significant, differences within the three supra-aortic BT measurement sites were present in the current study. During the phases with dynamic temperature changes (P2 and P4), the LoAs of the BT_nas_ were slightly higher than those of BT_tym_ and BT_ear_. Nasopharyngeal BT is considered a suitable technique to assess core BT during the process of invasive cooling after cardiac arrest^18,19^. The current findings indicate that in-ear BT measures could even deliver more robust body temperature data during fast temperature changes while mHCA. Both areas, the nasopharynx and the ear, share vascularization via the external carotid artery, so the discrete variation of the BT data might originate also by procedural differences. As a possible explanation, the employed in-ear sensor with its silicone head mold putatively offers thermal insulation for the ear canal, thereby protecting it against ambient temperature changes^20^ and manipulation effects. In this sense, the resulting continuous ear-canal insulation could generate a local microclimate, that might improve the quality of cBT estimation at this site^21^.

The results of this study do not support findings of former investigations, that indicate a systematic body temperature gradient in the ear canal with the warmest point at the tympanic membrane^22^. We found non-significant trends towards narrower LoAs and smaller biases of the BT_tym_ compared to BT_ear_ especially in phases of fast temperature changes. The latter might indicate a faster response of tympanic BT to changes in blood temperature due to different vascularization patterns between the ear-canal tissue and the tympanum^10^. Alternatively, different technical specifications (epitympanic: IR-thermography; ear canal: thermistor) of the in-ear sensor might explain the variation in the data.

### Body temperature estimation in the bladder and rectum during TTM/TH and mHCA

Rectal and bladder thermistor probes are commonly utilized techniques for temperature monitoring during surgical procedures and in intensive care, primarily due to its beneficial placement options.

During TTM/TH of mHCA, high agreements and high correlations of BT_ves_ and BT_rec_ with BT_eso_ were found under normothermic conditions^23^. At mHCA and during the cooling and rewarming of the patients, the bias of both body temperature measures exceeded 3 °C, with wide LoAs and only moderate ICC values.

Temperature change rates were significantly lower in BT_ves_/ BT_rec_ compared to BT_eso_, implying slower response dynamics during TTM/TH. In the hypothermic phase, bladder and rectal BT exhibit significant biases of + 3 to + 4 °C. This disparity constantly persisted over P3 without an obvious tendency to approach the body temperature estimations of the esophagus or the supra-aortic measurements. Therefore, this effect is probably not solely a result of a slower dynamic of BT_ves_/ BT_rec_ after the cooling process. Differences in tissue vascularization patterns and the ‘reservoir’ function of the bladder/rectum may account for this bias. Although not directly examined, the production of urine and its intraoperative drainage protocol influenced the bladder BT during our study procedures (data not shown).

Blood and tissue temperatures of the supra-aortic regions were significantly lower than those of the bladder and rectum during mHCA. On a single-patient basis, bladder/rectal BT was systematically higher and slower in response in comparison to supra-aortic BTs during all phases except for normothermia.

Former studies provide evidence for a good agreement of BT_ves_ and other cBT measurements in the context of mild TTM/TH (32–34 °C)^8^. This agreement putatively diminishes for mHCA with its fast BT gradients.

### Clinical applicability and generalizability of non-invasive cBT measurements

This study indicates a highly accurate core body temperature estimation at the epi-tympanum, the ear canal, and the nasopharynx during moderate hypothermia and rapid temperature changes in an intraoperative, well-controlled setting. Considering the minor invasivity of the in-ear measurement and its direct vascularization via carotid arteries, in-ear BT measurement procedures appear to be a useful adjunct in patient management during mHCA^24^. Epitympanic BT estimations with a preserved insulation of the ear canal might have beneficial effects on the quality of the estimation.

Conclusions regarding the generalizability of supra-aortic BT estimation procedures to monitor body temperature in other forms of hypothermia are limited. The fast temperature changes of the used TTM/TH protocol are normally higher than those during accidental hypothermia, although case reports of patients with accidental hypothermia (especially when extricated from avalanches or water), have already reported fast temperature drops up to 0.5 °C per minute^25^. Moreover, vasoregulation between TTM/TH and accidental, external hypothermia might be considerably different: TTM/TH originates by direct perfusion of cold blood through cannulated, central arteries. As a result, the BT derived from the local tissue depends on the temperature of the perfusate. In contrast, hypothermia caused by external cooling might induce significant differences in vasoregulation and local thermohomeostasis that can influence the BT estimations in the ear. There is evidence for effects on thermoregulation and perfusion dynamics of the ear induced by a decline of facial skin temperature^26^. In this line, studies in prehospital settings have highlighted the IR-thermography to produce unreliable results^27^. This could be a result of a high vulnerability to ambient and skin temperature changes^28^.

### Limitations of the study

The employed in-ear sensor provides a dual temperature estimation using IR-thermography (epitympanic) and thermistor (ear canal) technology, which might induce technical variation in the data. This limitation, however, was mitigated through direct warm-water calibration of the systems as described in the method section with an offset < 0.1 °C between the different BT measurement techniques.

Moreover, the findings have to be interpreted in the context of an optimally fitted ear/head ambiance and the induction of hypothermia via an internal stream of cold blood through cannulation and selected perfusion of central arteries. Herewith, HLM delivered blood flow to the ear canal with a targeted temperature with unclear consequences on vasoregulation of the supra-aortic circulation. Especially the effects of a trigeminovascular regulation, e.g. induced by changes of the skin temperature of the face, have to be further evaluated to increase the generalizability of in-ear BT measurements in the context of cBT monitoring during other clinical scenarios.

## Conclusions

This study supports the approach of using minimally invasive in-ear techniques to estimate core body temperature in an intrahospital TTM/TH setting of mHCA. Due to the common blood supply of the carotid arteries and the proximity of the ear to brain temperature, an estimation of brain temperature may be assumed, though this was not directly examined in our study.

## Supplementary Information

Supplementary Information.

## Data Availability

The datasets used and/or analysed during the current study are available from the corresponding author on reasonable request.
