# Supplementary material for: Agreement of in-ear temperature to core body temperature measures during invasive whole-body cooling for hypothermic circulatory arrest in aortic arch surgery
Source: Sci Rep. 2024 Nov 11;14:27607. doi: 10.1038/s41598-024-77237-5 (PMC11554646; doi:10.1038/s41598-024-77237-5)
Supplement: Supplementary file 1 — Supplementary Information. [file 41598_2024_77237_MOESM1_ESM.docx]

Standard perioperative monitoring during aortic arch surgery consists of central venous pressure, transoesophageal echocardiography and double- or triple-site invasive blood pressure measurement. The cerebral blood flow is routinely monitored noninvasively by near-infrared spectroscopy (NIRS). Procedures were performed via a full-median or partial-upper sternotomy. Arterial cannulation site for cardiopulmonary bypass were preferably direct aortic cannulation or, in selected and re-operative cases, the subclavian artery. The acid-base management followed the alpha-stat principles of temperature-uncorrected blood gas analysis and cardioplegic arrest is routinely initiated by ice cold Bretschneider’s crystalloid solution or mixed blood/crystalloid delNido solution. Standard for hypothermic arrest are moderate core body temperatures at 26°C, measured in the bladder, with bilateral selective antegrade cerebral perfusion at 22°C (cerebral perfusion pressure 50-60 mmHg; flow rate 6-10 ml/kg/min). Operative procedures included supracoronary ascending aorta replacement or root replacement in valve-sparing David or valve-replacing modified Bentall technique at proximal site. Distally, either the proximal arch including the entire inner curvature or the total arch with or without frozen elephant trunk technique was replaced.
